# Bone Allograft Prosthesis Composite to Revise a Failed Massive Allo-Prosthesis: Case Report and 10 Years of Follow-Up

**DOI:** 10.7759/cureus.12172

**Published:** 2020-12-19

**Authors:** Manuel R Medellin, Alejandro Abiad, Vanessa Salinas, Luis Carlos Gomez-Mier, Camilo Soto Montoya

**Affiliations:** 1 Orthopaedic Oncology, Instituto Nacional de Cancerología, Bogotá, COL; 2 Orthopaedic Oncology, Clinica Nueva, Bogotá, COL

**Keywords:** allograft prosthesis, bone allograft, osteochondral allograft, revision surgery

## Abstract

An 18-year-old male patient with a high-grade osteosarcoma was initially treated with resection and reconstruction using an osteochondral allograft. The allograft collapsed after five years, and thus a revision with a constrained knee prosthesis was performed. After one year, the implant failed due to a fracture, requiring another revision with a new allo-prosthetic composite. The long-term results were satisfactory.

Allo-prosthetic composites may offer good long-term results after sarcoma resection. The failure of a massive bone allograft does not preclude the use of another allograft to maintain the bone stock and preserve the function.

## Introduction

Limb preservation surgery is the currently the ideal option for the treatment after the resection of primary bone sarcomas [1]. Although the disease-free survival period after limb salvage does not change in comparison with amputation [2], the quality of life in all the patients improve in a significant manner [2]. Still today, there is discussion on the most adequate technique for reconstruction after the resection of bone tumors and several options are available, including: prosthetic replacement, implantation of allograft prosthesis composites (APCs), the use of allografts alone, and, in some cases, the temporal or definitive cement spacers.

All types of reconstructions have different advantages and disadvantages. The APCs were introduced with the aim of reducing the complications identified after the use of osteochondral allografts, such as fractures, arthrosis, and articular collapse [3]. In reconstruction using APCs, the articular surface of the allograft is replaced with a conventional or revision prosthesis, providing a more durable joint. The main advantage of this type of reconstruction is that once the allograft is integrated into the native bone, the load transfer between the prosthesis and the bone is similar to the one observed in a conventional replacement [4].

The APCs may avoid some complications observed with the use of mega-prosthesis, such as the concentration of stress at the union between the stem and the collar or the wear and loosening of the implant with subsequent bone loss [5]. However, the complications observed after the reconstruction with APCs may include loosening of the prosthesis, nonunion of the allograft, fractures, and infection [6].

In this report, we present the case of a patient initially managed with an osteochondral allograft, who presented a failure and was thus converted to an APC. This second reconstruction failed due to a fracture in the graft and stem, requiring a revision with a new APC. This last reconstruction has had an adequate medium- to long-term result.

## Case presentation

**Figure 1:**
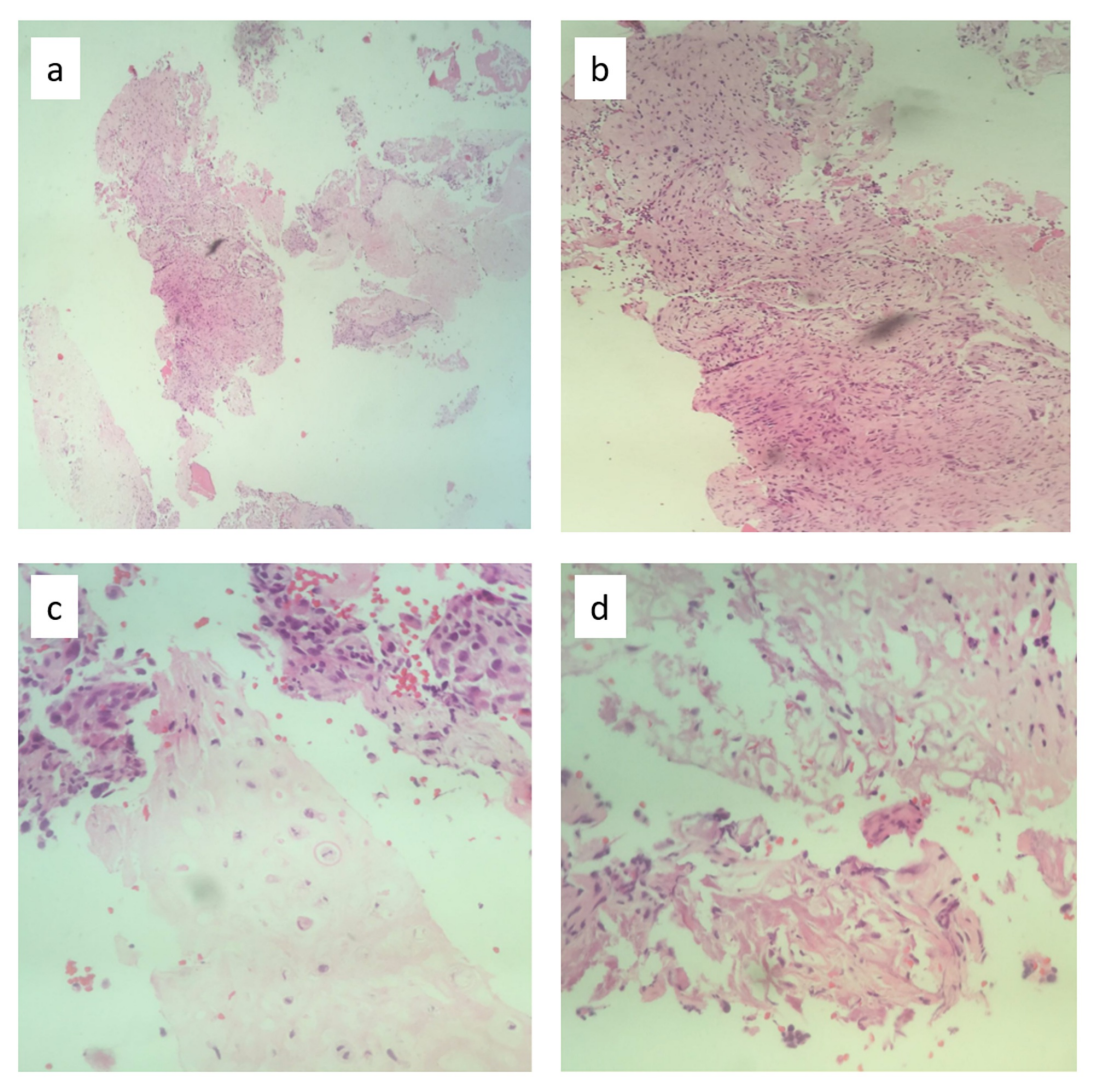
Hematoxylin-eosin pathology slides from the initial biopsy sample confirming a conventional osteoblastic and chondroblastic osteosarcoma. In (a), a low power field observation were two components can be identified, a chondral one and a very cellular one. In (b), at middle power field, high pleomorphic cellularity with increased atypical mithoses can be observed. In (c), at high power field, a high grade condroblastic component with some bipolar mitoses is identified. In (d), immature woven bone formation with atypical mitoses confirm also the osteoblastic component of the tumor.

An 18-year-old male patient presented with a history of mass and pain in the thigh. After the imaging, a biopsy was taken confirming the final diagnosis of a high-grade chondroblastic/osteoblastic osteosarcoma of the distal femur stage IIB (Figure 1).

The patient received neoadjuvant chemotherapy with MAP (methotrexate, doxorubicin, and cisplatinum), and limb salvage surgery was subsequently performed in October 2000. For the reconstruction, an osteochondral allograft fixed with a rigid stainless steel plate was selected. In the postoperative period, the extremity was immobilized with an articulated brace until some signs of union in the imaging were observed. The patient successfully completed the adjuvant chemotherapy course without evidence of recurrence or metastasis. The allograft unfortunately progressed to non-union, and after one year of the fixation (November 2001) a fracture in the plate was identified. The case was discussed and the decision was to revise the osteosynthesis. In the subsequent follow-up, weight-bearing was restricted until consolidation was achieved. During the rehabilitation process, an articulated brace in the knee was prescribed, with the association of progressive gain in quadriceps strength.

**Figure 2:**
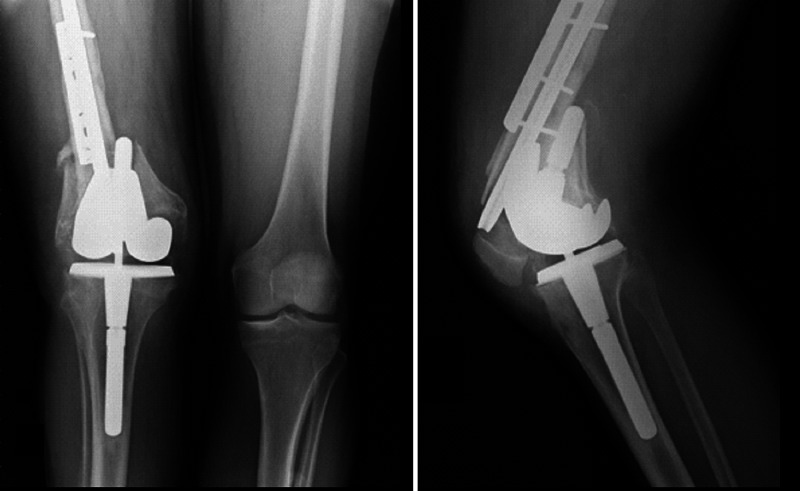
Anteroposterior and lateral knee X-rays taken two years after the second distal femoral allograft implantation. A supracondylar-intercondylar fracture is observed in association with the structural allograft failure.

One year after the last surgery (November 2002), the dislocation of the extensor mechanism and posteromedial femorotibial subluxation was identified. The range of movement at this time was acceptable (10° to 90°), and the pain was manageable; therefore, no further intervention was performed until January 2004, when the patient presented with pain. At this time, several osteoarthritic changes were observed, and for that reason the knee joint was replaced with a constrained revision implant. The results were satisfactory with early recovery in the range of movement (0°-100°), without leg length discrepancy and adequate strength to achieve gait without external aids. In November 2006 (two years after the last intervention), the patient presented to the clinic with sudden loss in the range of movement and with pain. New imaging identified a supracondylar fracture in the allograft, with the subsequent failure of the knee implant (Figure 2).

**Figure 3:**
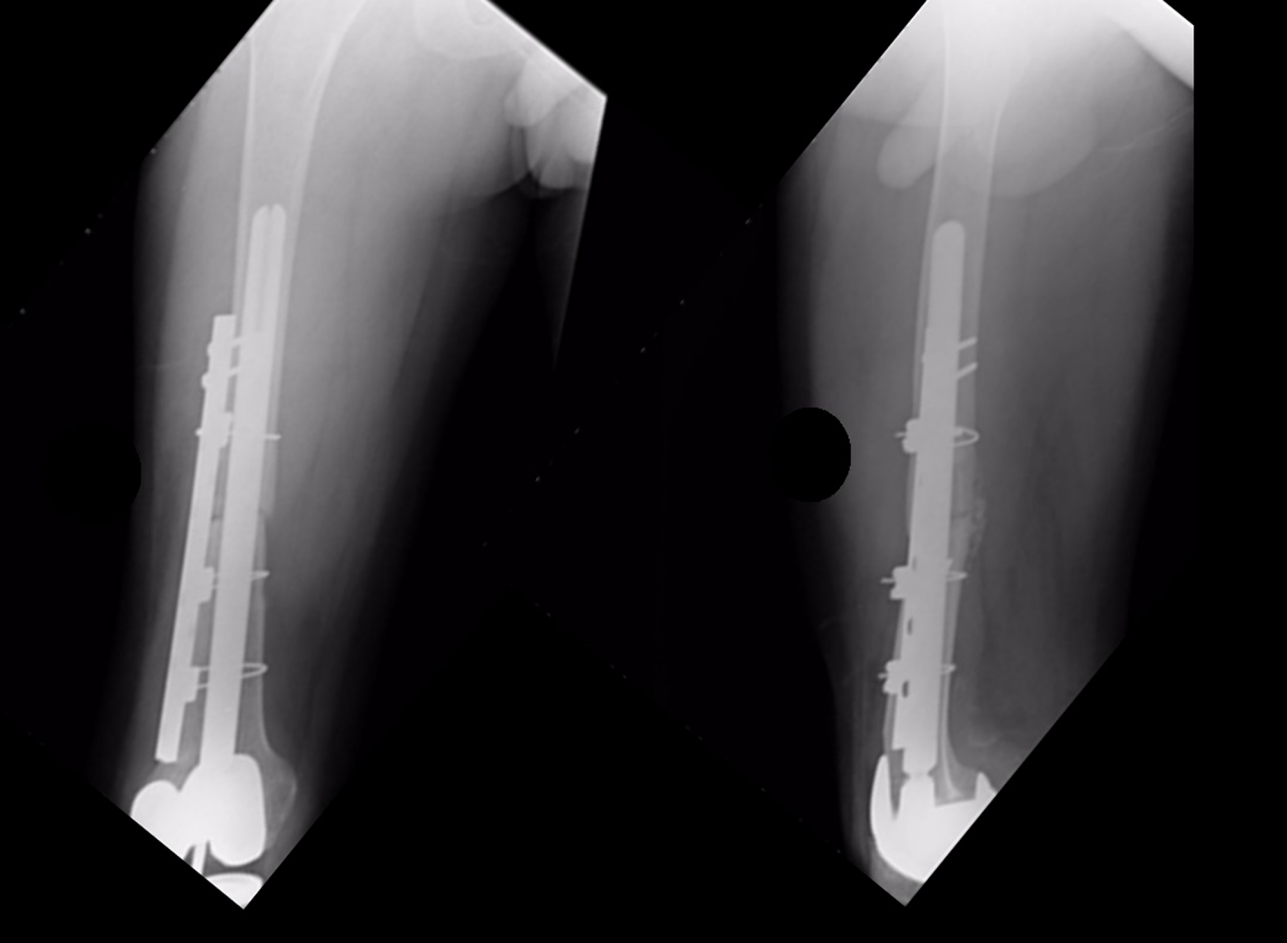
Anteroposterior and lateral X-rays of the femur in the postoperative period after the second distal femoral allograft prosthesis composite implantation. A long stem was selected to increase the stability of the new allograft. Additionally, a plate with cables reinforced the fixation.

After considering the options available, the therapeutic decision was to perform a revision with a new APC, as it was felt that an endoprosthetic replacement would lead to important bone loss. The patient had the new intervention in February 2007, in which the fractured allograft was removed and a new massive bone allograft was placed. The reconstruction was stabilized with a revision prosthesis with a long femoral stem (28 cm) and a 12-hole plate with cables (Figure 3).

**Figure 4:**
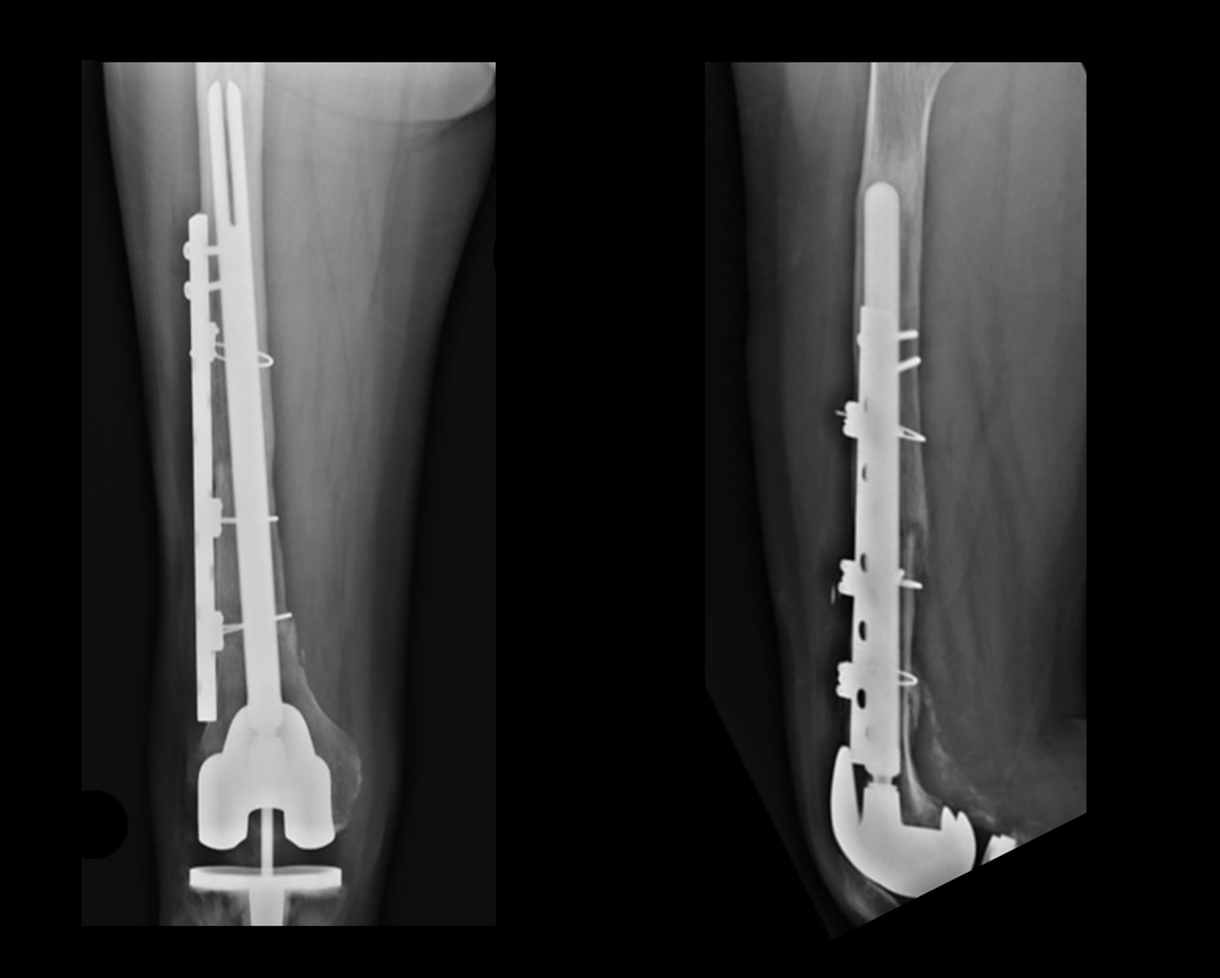
Anteroposterior and lateral X-rays of the femur taken 10 years after allograft revision. The allograft is integrated, stable, and well fixed. No pain is reported by the patient.

**Figure 5:**
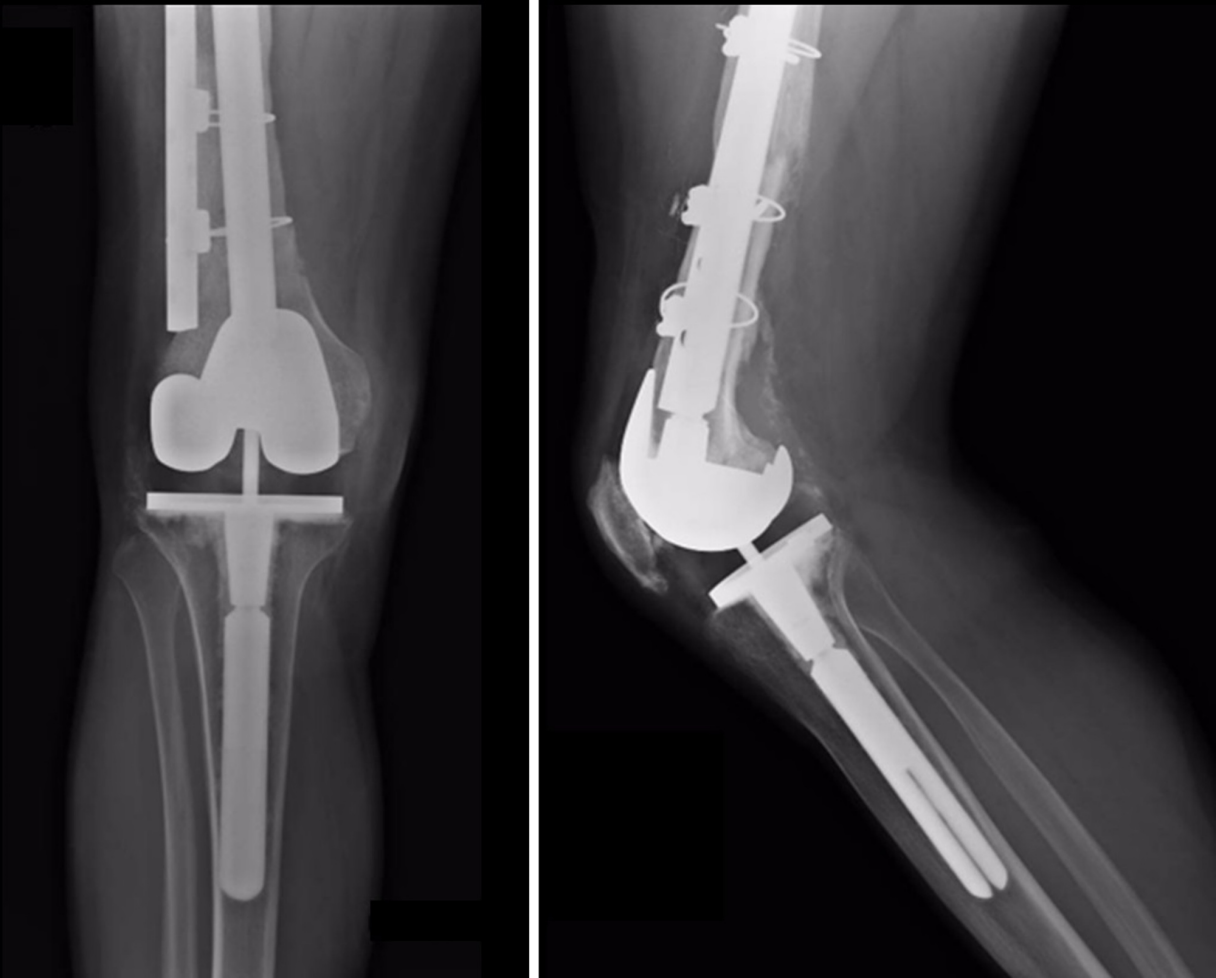
Anteroposterior and lateral knee X-rays taken 10 years after allograft revision. Revision knee implant is still stable. No problems have been recorded or reported by the patient.

The clinical progression was adequate, with a recovery of the movement and a decrease in the preoperative pain levels. During the follow-up, the integration of the bone graft was observed, with the maintenance of appropriate alignment and relationship between the prosthesis, the bone allograft, and the native bone (Figures 4, 5).

**Figure 6:**
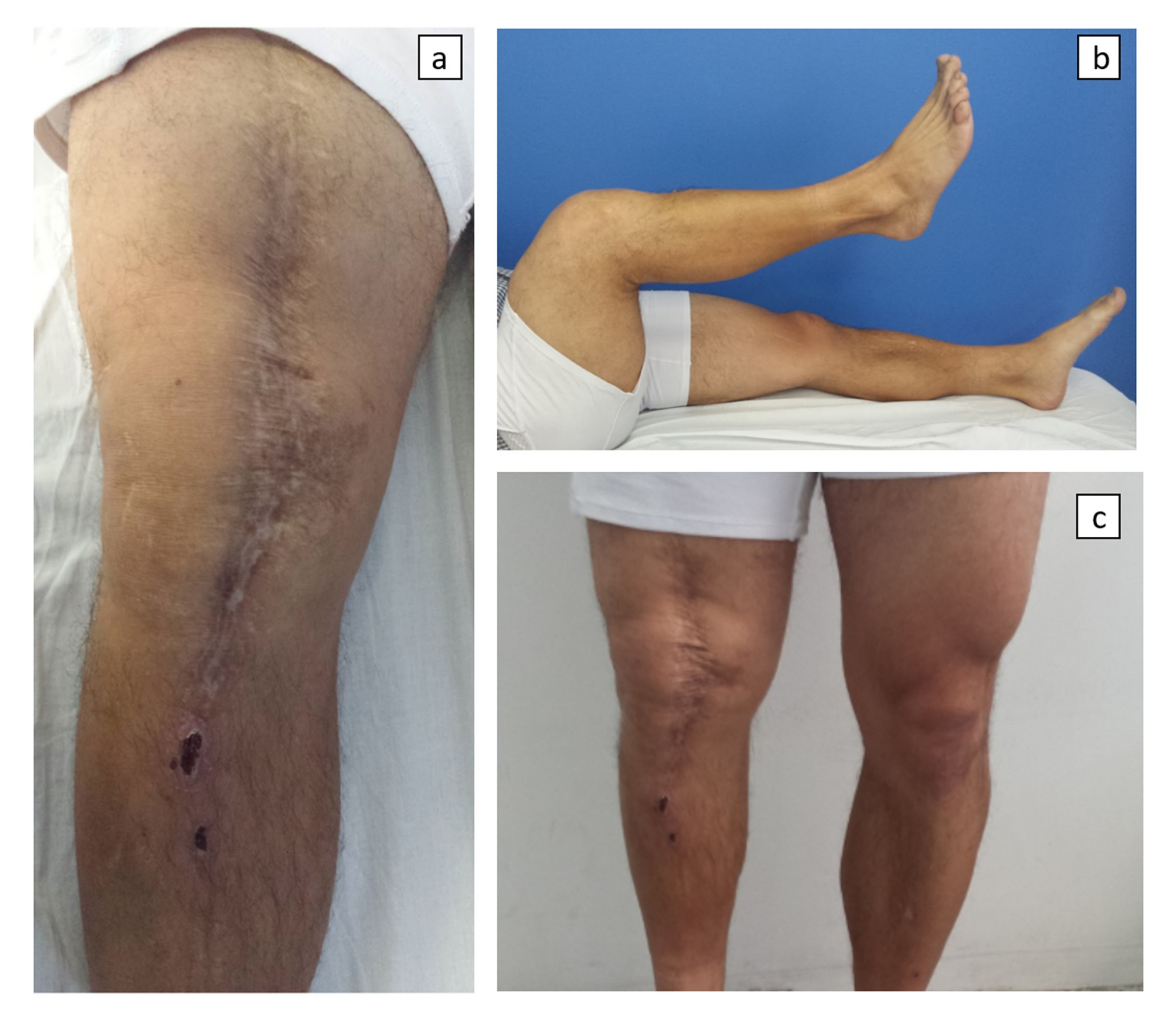
Clinical images of the patient at the last follow-up. (a) The knee is fully extended in the supine position. (b) Knee flexion in the supine position, presenting an adequate range of movement. (c) In the standing position, the extension of the knee is adequate, with a similar patellar level.

At present, the patient is 36 years old and has been followed for 10 years since the last intervention. He is able to walk without any external aids, with no leg length discrepancy. He can perform full extension of the knee and flexion up to 90° (Figure 6).

At the last visit to the clinic, no further surgical interventions have been required.

## Discussion

The most adequate method for reconstruction after massive bone loss and the best option to revise failed reconstructions are still controversial topics. In young patients with long life expectancy, massive bone allografts are an interesting option in order to preserve bone stock, restore the joint surface, and reattach stabilizing structures such as ligaments and tendons [5].

Using massive bone allografts is still challenging as several complications can be observed, such as fractures, delayed consolidation, non-union, hardware failures, osteoarthritis, and infection [6]. When the reconstruction is successful, long-term results can be achieved, with adequate functional results, but this requires from the surgeon a wide experience in reconstructive techniques and the familiarity with bone transfers and biologic reconstruction in order to recognize and avoid complications [7].

The long-term survival of massive allografts and allo-prosthetic composites has been presented by several series before [6,7], demonstrating that these reconstructions are suitable options after catastrophic bone loss [8,9]. In the literature, however, very little has been published regarding the revision of failed massive allografts and allo-prosthetic composites [10]. In a previous series by Aponte-Tinao et al. [4], an evaluation of the management of failed intercalary allografts was presented. In that series, it is suggested that fractures may be treated by replacing the graft with a new structural one when the bone involved is femur and non-union is observed. In that paper, one of the failures was treated with an osteochondral allograft in one patient. Sorger et al. [11] described a very long series of patients with fractured allografts. The reconstruction of failed osteochondral allografts with allograft prosthetic composites is presented in that paper, but no reference is made to failed allograft prosthetic composites.

In our study, we present a possible solution after failed allograft-prosthesis composites. To the extent of our knowledge, this is the first description of the revision of an allograft prosthetic composite with a new allograft and prosthesis with good medium- to long-term functional results. As it has been presented by several publications, rigid fixation is preferable in order to increase the chances of integration at the junction. In our case, we decided to use a plate and cables instead of screws to keep stable the allograft. We believe this fixation was complimentary, as the rest of the allograft was anchored by the implant stem. We believe that the use of screws would have increased the chance of fractures without any additional benefit, but the type of fixation in reconstructive surgery depends on the particular conditions of every case.

Endoprosthetic replacement is often selected as the method of treatment for failed allografts. Although endoprosthetic replacements offer good long-term functional results, we feel that factors such as longevity of the implant, the high cost of the prosthetic components, the increase in bone loss if revision is required, and the impossibility to reattach/reconstruct the soft tissues may prevent their use in all revision cases.

## Conclusions

In conclusion, the history of multiple revision surgeries that included allograft prosthetic composites does not preclude the use of a new allo-prosthesis in limb preservation surgeries. This case exemplifies the good long-term results that can be obtained when using this method of reconstruction especially in young and active patients who may develop more complications with an endoprosthetic replacement.
